# Modulation of *Escherichia coli* Translation by the Specific Inactivation of tRNA^Gly^ Under Oxidative Stress

**DOI:** 10.3389/fgene.2020.00856

**Published:** 2020-08-18

**Authors:** Lorenzo Eugenio Leiva, Andrea Pincheira, Sara Elgamal, Sandra D. Kienast, Verónica Bravo, Johannes Leufken, Daniela Gutiérrez, Sebastian A. Leidel, Michael Ibba, Assaf Katz

**Affiliations:** ^1^Programa de Biología Celular y Molecular, ICBM, Facultad de Medicina, Universidad de Chile, Santiago, Chile; ^2^Department of Microbiology and The Center for RNA Biology, The Ohio State University, Columbus, OH, United States; ^3^Max Planck Research Group for RNA Biology, Max Planck Institute for Molecular Biomedicine, Münster, Germany; ^4^Cells-in-Motion Cluster of Excellence and Faculty of Medicine, University of Münster, Münster, Germany; ^5^Research Group for RNA Biochemistry, Department of Chemistry and Biochemistry, University of Bern, Bern, Switzerland; ^6^Unidad de Microbiología, Escuela de Medicina, Facultad de Ciencias Médicas, Universidad de Santiago de Chile, Santiago, Chile

**Keywords:** tRNA^Gly^, *Escherichia coli*, oxidative stress, codon, glycine, translation

## Abstract

Bacterial oxidative stress responses are generally controlled by transcription factors that modulate the synthesis of RNAs with the aid of some sRNAs that control the stability, and in some cases the translation, of specific mRNAs. Here, we report that oxidative stress additionally leads to inactivation of tRNA^Gly^ in *Escherichia coli*, inducing a series of physiological changes. The observed inactivation of tRNA^Gly^ correlated with altered efficiency of translation of Gly codons, suggesting a possible mechanism of translational control of gene expression under oxidative stress. Changes in translation also depended on the availability of glycine, revealing a mechanism whereby bacteria modulate the response to oxidative stress according to the prevailing metabolic state of the cells.

## Introduction

Bacteria, like other organisms, need to adapt to environmental conditions that are constantly changing. Some of these conditions induce oxidative stress in bacteria due to either an increase in oxidants or a decrease in the ability of bacteria to defend against them. In the absence of an adequate protective response to oxidative stress, numerous macromolecules may be damaged, including proteins, lipids, and nucleic acids (Imlay, 2008).

The response to oxidative stress has been extensively studied, in particular because generation of an oxidative attack by macrophages and polymorphonuclear leukocytes is one of the main defense strategies of the human body against invading bacteria once they have crossed the primary physical barriers (Slauch, 2011; Nguyen et al., 2017). In order to adapt to conditions that induce oxidative stress, bacteria may: (I) reduce motility, increase exopolysacharide production, and induce biofilm formation, thereby reducing accessibility to molecules that produce oxidative stress (Gambino and Cappitelli, 2016); (II) inhibit replication, preventing DNA mutagenesis at sites of base oxidation (Imlay, 2013) and possibly also reducing the toxicity of replication near the site of repair for oxidized bases (Charbon et al., 2014); (III) reduce the rate of global translation (Katz and Orellana, 2012; Zhong et al., 2015; Zhu and Dai, 2019); (IV) selectively induce the production of several proteins involved in the reduction, repair, or degradation of oxidant molecules or oxidized biological targets such as thiol groups and Fe/S clusters in proteins (Imlay, 2013); and (V) decrease the production of reduced nicotinamide adenine dinucleotide (NADH) in order to increase production of reduced nicotinamide adenine dinucleotide phosphate (NADPH), required to reduce oxidant molecules and oxidized targets (Rui et al., 2010; Shen et al., 2013). Finally, oxidative stress also induces some members from a bacterial community to enter a partially quiescent state known as “persistence” (Wu et al., 2012), where several primary metabolic pathways are repressed and stress responses are induced (Lewis, 2010; Cohen et al., 2013).

Transcriptional control of gene expression plays a major role in coordinating the cellular response to oxidative stress. In *Escherichia coli* and other enterobacteria, OxyR and SoxR sense the presence of hydrogen peroxide and oxygen superoxide, respectively, regulating the transcription of genes that belong to each regulon (Imlay, 2013). These transcription factors are aided by others, like Fur or Fnr, that modulate more specific aspects of the response (Chiang and Schellhorn, 2012; Shimizu, 2016). Beyond this “transcription focused” response, reports from diverse laboratories suggest that the defense mechanisms against oxidative stress also depend on translation, for example, *via* regulation of transfer RNA (tRNA) metabolism and translation elongation. It has been shown that changes in error rate of aminoacyl-tRNA synthetases are altered by oxidation of editing domains that may increase or decrease their activities under oxidative stress (Ling and Söll, 2010; Wu et al., 2014; Steiner et al., 2019). Others have found that tRNA may be oxidized (Liu et al., 2012) and, in at least some *E. coli* strains, oxidative stress induces a general and undiscriminated degradation of tRNAs that strongly reduce translation elongation, eventually provoking cell death (Zhong et al., 2015; Zhu and Dai, 2019). Others have found that, in *E. coli* and other bacteria, oxidation of translation elongation factors may also inhibit elongation (Kojima et al., 2009; Nagano et al., 2015; Yutthanasirikul et al., 2016).

To investigate whether changes in the concentration of particular tRNAs may modulate the translation of the *E. coli* transcriptome, we screened for changes in the levels of functional tRNAs under sub-lethal oxidative stress. Unexpectedly and in contrast to other reports, we observed that in the strain used in this research, *E. coli* K-12 MG1655, only tRNA^Gly^ is inactivated under oxidative stress while nine other tRNAs remain active and at unchanged concentrations. Over production of specific tRNA^Gly^ isoacceptors altered the response of *E. coli* to oxidative stress, altering motility, carbohydrate consumption, and growth kinetics. The observed changes directly correlated with alterations in the translation efficiency of Gly codons under oxidative stress, suggesting a mechanism by which changes in active tRNA^Gly^ levels may regulate the response to oxidative stress.

## Materials and Methods

### Strains and Culture Media

All experiments performed in this work used wild-type (WT) *E. coli* K-12 MG1655 strain. Strains were cultured in either lysogeny broth (LB) media (1% tryptone, 0.5% yeast extract, and 0.5% NaCl), M9 media (47.7 mM Na_2_HPO_4_, 22.0 mM KH_2_PO_4_, 8.6 mM NaCl, 18.7 mM NH_4_Cl, 2 mM MgSO_4_, 0.1 mM CaCl_2_, and 0.4% Glycerol), or low phosphate media (40 mM MOPS, 4 mM Tricine, 50 mM KCl, 10 mM NH_4_Cl, 0.2 mM KH_2_PO_4_, 0.5 mM MgSO_4_, 10 μM FeCl_3_, and 0.4% Glucose) supplemented with branched amino acids. When indicated, isopropyl β-d-1-thiogalactopyranoside (IPTG; 100 μM), branched amino acids (isoleucine, leucine, and valine 50 μg/ml each), Gly (50 μg/ml glycine), diverse sugars (glucose, arabinose, lactose, or manose, 0.2–0.4%), phenol red (0.1 μg/ml), ampicillin (100 μg/ml), or paraquat (up to 1M) were added to the culture media.

### tRNA Purification

tRNA was extracted using previously published protocols (Raczniak et al., 2001) with increased 2-mercaptoethanol added to quench possible remnants of oxidizing molecules. 250 ml of LB were inoculated with 1.5 ml of an overnight culture of *E. coli*, incubated at 37°C, and shaken at 225 rpm. At an OD_600_ of 0.6–0.7, H_2_O_2_ or paraquat was added for a final concentration of 2.5 and 1 mM, respectively. After 20 min (H_2_O_2_) or 30 min (paraquat) of incubation, the culture was pelleted at 11,000 *g* for 5 min at 37°C. Cells were resuspended in 2.5 ml of buffer A (20 mM Tris HCl, pH 7.0; 20 mM MgCl_2_; and 20 mM 2-mercaptoethanol) and extracted by shaking for 20 min at room temperature with 2.5 ml of acid phenol. Aqueous phase was recovered after centrifugation at 6,500 *g* for 10 min at room temperature and stored at 4°C. Phenol was re-extracted with additional 2.5 ml of buffer A. Both buffer A extracts were mixed together and re-extracted with 5 ml of acid phenol. The aqueous phase was recovered. Isopropanol was added to a final concentration of 20% and was centrifuged at 9,000 *g* for 60 min. The supernatant was recovered and isopropanol concentration was adjusted to 60%. The mixture was centrifuged at 11,000 *g* for 60 min. Supernatant was discarded, the pellet briefly dried and then dissolved in 1.25 ml of 200 mM Tris acetate pH 8.5 plus 20 mM 2-mercaptoethanol and incubated for 60 min at 37°C to deacylate tRNAs. Samples were further purified in a DE52 or DEAE sepharose column (~250 μl resin). Sample was loaded in the column and subsequently cleaned with 50 volume buffer II (20 mM Tris HCl pH 7.0; 200 mM NaCl and 5 mM 2-mercaptoethanol). tRNA was eluted with buffer III (20 mM Tris HCl, pH 7.0; 1M NaCl; and 5 mM 2-mercaptoethanol). tRNA from fractions with higher absorbance at 260 nm was recovered by precipitating with 0.1 volume 3 M sodium acetate pH 4.5 and 2 volume ethanol. Samples was stored at −20°C for at least 30 min and then centrifuged at 9,000 *g* for 1 h. Pellets were cleaned with cold 80% ethanol and resuspended in H_2_O.

tRNA samples used for mass spectrometry analyses were further purified using biotinylated beads. For these samples, 180 μg of total tRNA were dissolved in hybridization solution [0.1× saline sodium citrate (SSC) buffer, 0.1% sodium dodecyl sulfate (SDS), and 5 mM 2-mercaptoethanol] containing 0.5 mM EDTA and 2.5 μM of the corresponding biotinylated probe. We used the same biotinylated probes as for Northern blots (Supplementary Table S3). The mixture was incubated for 2 min at 90°C and then rapidly cooled to 41°C. Samples were further incubated for 120 min at this temperature. Samples were then mixed with 60 μl streptavidin/sepharose beads (in 150 μl hybridization buffer) and incubated for 30 min at this temperature while shaking at 1 min intervals. Samples were centrifuged for 20 s at 3,000 *g* and the supernatant was eliminated. Then samples were cleaned eight times with hybridization buffer (3 min incubations at 41°C with gentle shaking each min. Spin 20 s at 3,000 *g* to eliminate supernatant) and eluted in 40 μl of the same buffer at 80°C.

### tRNA Quantification by Aminoacylation

tRNA’s concentration was estimated from the plateau of an aminoacylation reaction progress curve at 37°C. Reactions were started by adding tRNA extracts to get 0.2 μg/μl in a mixture containing 1× reaction buffer (100 mM HEPES KOH, pH 7.2; 30 mM KCl; and 12 mM MgCl_2_), 5 mM ATP pH 7.0, 10 mM 2-mercaptoethanol, 8 U/ml pyrophosphatase (Roche 10 108 987 001), 2.7 mg/ml of a S100 extract from *E. coli* K-12 MG1655 (cleaned using DE52 resin to eliminate amino acids and RNA), and a mix of non-radioactive and ^14^C amino acid (Supplementary Table S4 for final concentrations). At defined time points, 7 μl of aliquots were deposited in filter paper saturated in 5% trichloroacetic acid to precipitate aminoacyl-tRNAs. Papers were washed at room temperature three times for 5 min in 5% trichloroacetic acid and once in 100% ethanol. Then, papers were dried and aminoacyl-tRNAs were quantified in a scintillation counter. Background was subtracted based on experiments where no tRNA was added to account for non-specific binding of radioactive amino acids to filter papers and the potential tRNA traces present from S100 extracts.

### RNA Mass Spectrometry

The detailed protocol for the analysis of RNA by mass spectrometry has been described elsewhere (Sarin et al., 2018). In brief, tRNAs were digested to single nucleosides essentially, as previously described (Alings et al., 2015). Chromatographic separations of the samples were performed using a self-packed Hypercarb capillary column (75 μm ID × 500 mm) coupled to a Proxeon EASY nLC (Thermo Fisher Scientific GmbH, Dreieich, Germany). Samples were separated using solvent A (5 mM ammonium formate pH 5.2) and solvent B (acetonitrile) in a multi-step gradient (2–20% B for 3 min; 20–75% B for 3–50 min; 75–100% B for 5 min; hold at 100% B for 15 min). Subsequently, samples were analyzed using a Q Exactive Mass Spectrometer (Thermo Finnigan LLC, San Jose, CA).

### Quantitative Analysis of LC-MS/MS Data

Thermo RAW files were converted to the mzML format (Martens et al., 2011) using msConvert as part of ProteoWizard (version 3.0.10738; Chambers et al., 2012). Quantitative data analysis was performed using pymzML (version 2.0.0; Bald et al., 2012) and pyQms (version 0.5.0; Leufken et al., 2017). Chemical formulae of all nucleosides (including modified forms) were retrieved from the MODOMICS database (Boccaletto et al., 2017).

pyQms was used to calculate high-accuracy isotopologue patterns for all chemical formulas, and these patterns were matched onto all MS1 spectra. Quantification of nucleosides for individual samples is based on the maximum intensity of the matched isotope pattern chromatogram (MIC). To assess quantification quality, pyQms calculates a weighted similar match score (mScore; Leufken et al., 2017). Detection and quantitation of selected nucleosides were manually validated.

### Total RNA Extraction

2 ml of bacterial cultures in LB or 5 ml from cultures in M9 media were pelleted for 1 min at 12,000 *g*. Pellet were resuspended in 50 μl lysis buffer (83 mM Tris HCl, pH 6.8, 18 mM EDTA pH 8, 1.7% SDS, and 1.6% 2-mercaptoethanol) and incubated for 3 min at 37°C. 1.5 ml of TRIzol was added, and total RNA was extracted following the manufacturer’s protocol.

### Northern Blot Assay

Most Northern blot analyses were performed using biotinylated probes. For some RNA, we additionally used a non-labeled probe to help “unwind” the tRNA structures (list of probes in Supplementary Table S3). Samples were transferred to positively charged nylon membranes for 2 h at 22 volts in 0.5× TBE. Then, RNA was fixated by UV radiation and membranes were blocked for 30 min at the temperature indicated in Supplementary Table S3 in hybridization solution (6× SSC, 70 μg/ml heat-denatured salmon sperm DNA, 0.1% SDS, and 5× Denhardt’s solution). After blocking, probes were added directly to the same solution and incubated overnight at the same temperature. Membrane was then washed for 3 min at room temperature with solution A (2× SSC and 0.1% SDS) and then twice for 15 min at the temperature specified in Supplementary Table S3 in solution B (0.1× SSC and 0.1% SDS). After this treatment, the membrane was blocked for 30 min at room temperature with a blocking solution [1% casein in maleic buffer (0.1M maleic acid and 0.15M NaCl pH 7.5)]. Then, 0.1 μg/ml of streptavidin-horseradish peroxidase was added to the blocking solution. Membranes were incubated for 30 min at room temperature and then washed twice for 15 min with maleic acid buffer plus 0.3% (V/V) tween-20. Finally, the membranes were washed for 3 min in pre-detection buffer (0.1M Tris HCl, 0.1M NaCl, pH 9.5) and developed using a chemiluminescent kit (SuperSignal West Pico Chemiluminescent Substrate, Prod#34080). Determination of aminoacylation levels in strains overproducing tRNAs was performed using ^32^P labeled probes. A similar protocol was used, but images were acquired using phosphorimager technology.

### Determination of the *in vivo* Levels of tRNA Aminoacylation

Total RNA was purified in acidic conditions, and then, the 3' extreme nucleotide was eliminated by sodium periodate oxidation followed by β-elimination following previously described protocols (Salazar et al., 2004). Briefly, 50 ml of LB were inoculated with 300 μl on preinoculum of *E. coli* and incubated at 37°C and constant shaking. At an OD_600_ of 0.9–1.0, 15 ml of culture was pelleted at 10,000 *g* for 6 min. Paraquat to 1 mM was added to the remaining culture and continued incubating for 30 min, after which 15 ml were similarly pelleted. Immediately after pelleting, each bacterial pellet was resuspended in 500 μl of 0.3 M sodium acetate pH 5.2 with 1 mM EDTA pH 8.0. After resuspension, 500 μl of acid phenol were added and the mixture was incubated for 10 min on ice with intermittent mixing. Then, phases were separated by centrifuging for 6 min at 10,000 *g*. Aqueous phase was recovered and RNA was precipitated by adding 1 ml of ethanol and storing samples at −80°C. After samples from stressed cells were kept for 30 min at −80°C, all samples were centrifuged (14,000 *g*, 30 min). Pellets were washed with 0.5 ml of 75% ethanol with 10 mM sodium acetate pH 5.2 and then resuspended in 50 μl of H_2_O. It is recommended not to use freshly distilled water to allow pH of water to decrease by absorption of atmospheric CO_2_. Each sample was divided into two 25 μl aliquots. 1.42 μl of 3 M sodium acetate of pH 5.2 was added to tubes “A” that were then stored at −80°C. tRNA in tubes “B” was deaminoacylated by adding 6.25 μl of 1 M Tris acetate pH 9.0 and incubating for 60 min at 37°C. Samples in tubes B were precipitated by adding 3.13 μl of 3 M sodium acetate of pH 5.2 and 62.5 μl ethanol. Samples were stored at −80°C for at least 30 min, after which samples were centrifuged (30 min, 13,000 rpm). Pellets were washed with ethanol 70%, dried, and resuspended in 26.4 μl of 160 mM sodium acetate pH 5.2. Samples A were thawed and 4.84 μl of freshly prepared 250 mM sodium periodate was added to tubes A and B. Tubes were wrapped in aluminum foil and incubated for 90 min on ice. Then, 12.97 μl of 20% glucose was added. After an additional 90 min incubation in ice, 4.3 μl of 3 M sodium acetate pH 5.2 and 87 μl ethanol were added. Samples were stored at least for 30 min at −80°C and centrifuged (30 min, 13,000 rpm). Pellets were resuspended in 250 μl of 0.5M lysine pH 8.0 and incubated for 60 min at 45°C. Then, 25 μl of 3M sodium acetate pH 5.2 and 500 μl ethanol were added, and samples were stored at least for 30 min at −80°C. Tubes were then centrifuged (30 min, 13,000 rpm), and after washing with 70% ethanol, pellets were dried and resuspended in 15 μl water. Then, samples were analyzed by 10% polyacrylamide gels with 8 M urea and by Northern blot analysis.

### Cloning and Mutation Protocols

Plasmid pBAD30SFIT (Rojas et al., 2018) contains a tandem fluorescent transcriptional fusion cassette composed of superfold green fluorescent protein (sfGFP) followed directly by a modified mCherry, itag-mCherry (Henriques et al., 2013). The plasmid contains a XhoI-SpeI site after the third codon of *sfgfp*, where tetra codon sequences were inserted using annealed oligo cloning with the oligonucleotide pairs described in Supplementary Table S5. *narJ* was also cloned in XhoI-SpeI restriction sites after amplifying the gene from *E. coli* K-12 MG1655 genomic DNA using external oligonucleotides NarJ_EcoRI_5'_Fw and NarJ_XhoI_3'_GGA_Rv (Supplementary Table S6). To clone the mutant version of *narJ*, a similar protocol was used, exchanging primer NarJ_XhoI_3'_GGA_Rv by NarJ_XhoI_3'_GGC_Rv. The same protocol was used to clone these genes in pBAD30SFIT-HP, which codes for a hairpin between *gfp* and *mCherry*. The hairpin was introduced in pBAD30SFIT by annealed oligo cloning of oligonucleotides Str_Yam_Fw and Str_Yam_Rv (Supplementary Table S6) in BglII y PciI restriction sites.

Cloning of tRNA genes was performed by annealed oligo cloning of oligonucleotides listed in Supplementary Table S7 between EcoRI and HindIII sites of pKK223-3.

*E. coli* K-12 MG1655 Δ*glyVX*::FRT was constructed by the Red-swap method (Datsenko and Wanner, 2000) using primers glyV (H1 + P1) and glyX (H2 + P2; Supplementary Table S8), as well as plasmid pLZ01 (Blondel et al., 2013) as template for amplification of a Cam resistance cassette flanked by the FRT sites (FLP recombinase target sequence).

### Translation Efficiency Analyses

M9 media supplemented with branched amino acids (50 μg/ml each) and ampicillin (100 μg/ml) were inoculated with bacteria from a saturated overnight culture in similar media and grown at 37°C in an orbital shaker. When indicated, the inoculated media were also supplemented with Gly (50 μg/ml). When bacteria reached mid-log phase (OD_600_ ~0.4–0.6), a 50 μl aliquot of it was diluted in a 96-well optical-bottom plate with 150 μl fresh M9 media supplemented with arabinose (0.4% final concentration). When indicated, media additionally contained paraquat (700 μM final concentration). Plates were further shaken for 2 h at 37°C. Then, OD_600_ and fluorescence intensity of green fluorescent protein (GFP; Ex. 480 ± 4.5 nm, Em. 515 ± 10 nm) and mCherry (Ex. 555 ± 4.5 nm, Em. 600 ± 10 nm) were measured in a microplate reader (Infinite M200 PRO, Tecan). When comparing GFP/mCherry fluorescence ratios of different strains (WT vs. Δ*glyVX*::FRT), data were normalized by the GFP/mCherry fluorescence ratio of a control without additional codons (S1).

### Motility Assay

Strains were cultured in LB media supplemented with 100 μg/ml ampicillin and 100 μM IPTG. When the culture reached an OD_600_ of ~0.7, an aliquot was centrifuged and the pellet was resuspended in LB to an OD_600_ of 1. 5 μl of these samples were used to inoculate LB plates with 0.3% agar, 100 μg/ml ampicillin and 100 μM IPTG. When indicated, 500 μM paraquat were also added to the plates. The plates were incubated during 8 (control) or 24 h (paraquat) in a humid chamber at 30°C. After this time, the radial growth was measured (Ha et al., 2014) and expressed as a ratio to colony diameter of the strain carrying the empty plasmid (62 ± 5 mm under control conditions and 19 ± 10 mm when 500 μM paraquat was added). These experiments could not be performed in plates with M9 media because motility was too low for all strains when paraquat was added.

### Growth Curves

*E. coli* K-12 MG1655 was transformed with plasmids carrying the genes for each tRNA^Gly^ isoacceptor and stored at −80°C. A fresh ON culture of these strains in LB media supplemented with 100 μg/ml ampicillin and 100 μM IPTG was diluted to DO_600nm_ ~0.05 in a similar media with or without 1 mM paraquat and transferred to a 96-well microtiter plate. Growth was subsequently followed in a thermostated microplate reader (Infinite M200 PRO, Tecan) at 37°C and intercalating orbital (142 rpm, 6 mm amplitude) and linear (296 rpm, 6 mm amplitude) shaking in 10 min intervals. Using strains that have been cultured several times in LB plates gave inconsistent results, suggesting that strains overproducing tRNA^Gly^ accumulate mutations that altered their behavior.

### Carbohydrate Fermentation Assay

ON cultures of bacteria grown in M9 media supplemented with glycerol 0.4%, Gly 50 μg/ml, branched amino acid of 50 μg/ml, and ampicillin 100 μg/μl were used to inoculate similar media and incubated at 37°C. When cultures reached an OD_600_ value of 0.6–0.8, a sample was diluted to an OD_600_ of 0.2 in similar media. Around 50 μl of these samples were mixed in a 96-well plate with 50 μl of similar media plus the corresponding carbohydrate (0.4% final concentration), IPTG (100 μM final concentration), and phenol red (0.1 μg/L) as pH indicator. Media also had diverse concentrations of paraquat (0–350 μM). Samples were incubated for 12 h at 37°C, after which plates were centrifuged (3,000 rpm, 7 min). Absorbance of the supernatant was measured at 560 nm.

### Paraquat MIC Determination

MIC values were estimated using previously described methods (Wiegand et al., 2008). Briefly, 50 μl of culture with 10^8^ CFU in M9 media supplemented with glycerol 0.4%, Gly 50 μg/ml, branched amino acids of 50 μg/ml each, 100 μM IPTG, and 100 μg/ml ampicillin were mixed with 50 μl of the same media containing serial dilutions of paraquat in 96-well plates. Plates were incubated in ON at 37°C, and then bacterial growth was determined by OD_600_.

### Analysis of Codon Usage

Codon usage for each gene was calculated with an in-house Perl 5 script and using the current RefSeq annotation for *E. coli* K-12 MG1655 genes (RefSeq assembly accession: GCF_000005845.2). Enrichment analysis was performed in EcoCyc platform (Keseler et al., 2017).

## Results

### Oxidative Stress Induces a Decrease in the Levels of Active tRNA^Gly^

**Figure 1:**
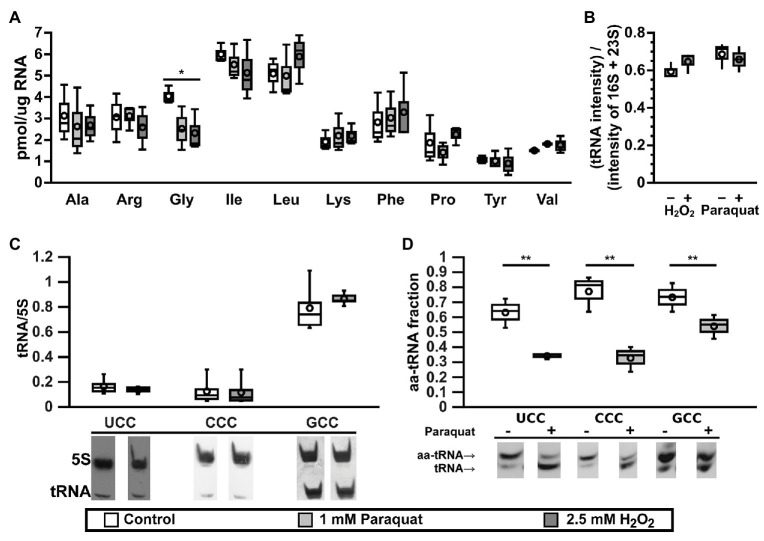
tRNA^Gly^ are inactivated under oxidative stress. **(A)** Effect of oxidative stress on the levels of active tRNAs specific for 10 different amino acids as measured by the plateau of aminoacylation reaction. Control: lysogeny broth (LB), white bars; oxidative stress: LB + 1 mM paraquat, light gray bars; or 2.5 mM H_2_O_2_, dark gray bars. ^*^p ≤ 0.01, one way ANOVA with Dunnett versus Control posterior test for each amino acid (*n* = 3). **(B)** Effect of oxidative stress on the levels of total tRNA from cells cultured in M9 media under control (white bars) or oxidative stress (gray bars, left: 2.5 mM H_2_O_2_ or right: 1 mM paraquat) and analyzed by electrophoresis (*n* = 3). Intensities from tRNA are higher than expected, as ribosomal RNA (rRNA) should represent at least 80% of total RNA. We suggest this is an artifact of staining efficiency and should not alter the conclusions of the figure. **(C)** Effect of paraquat on the levels of tRNA as quantified by Northern blot of total RNA samples purified from control (LB, white bars) or stressed (LB + 1 mM paraquat, gray bars) *E. coli* cells. Data in the graph showed no significant differences using one way ANOVA (*n* = 5). **(D)** *In vivo* levels of aminoacylation of tRNA^Gly^ in *E. coli* cell before (white bars) or 30 min after stress by 1 mM paraquat (gray bars). 3' terminal nucleotide of RNAs was eliminated by oxidation with sodium periodate followed by β-elimination and analyzed by Northern blot. ^**^p ≤ 0.05, paired *t*-test (*n* = 3). In all box graphs, top, middle, and bottom lines of the box represent 25, 50, and 75% of the population. Whiskers represent the maximum and minimum values and the mean is represented by a circle.

Aminoacylated tRNAs (aa-tRNAs) are essential for elongation of the nascent peptide during mRNA translation. While translation is mainly regulated at the initiation step, changes in the modification status and/or aminoacylation levels of tRNA can regulate translation by altering elongation rates (Starzyk, 1984; Subramaniam et al., 2013a, 2014; Katz et al., 2016; Zhu and Dai, 2019). To determine the role of tRNA alterations in the bacterial oxidative stress response, we screened for changes in concentrations of active tRNAs upon exposure to oxidants using the tRNA aminoacylation reaction. Total tRNA was purified from *E. coli* K-12 MG1655 (Blattner et al., 1997) cells cultivated under control conditions or oxidative stress induced by addition of 1 mM paraquat or 2.5 mM H_2_O_2_. Addition of paraquat led to a minor decrease in growth, while exposure to H_2_O_2_ arrested cell growth for ~2 h, after which cells resumed replication (Supplementary Figure S1). Purified total tRNA was deaminoacylated and subsequently selectively aminoacylated with 10 available radioactive amino acids using cell-free extracts from *E. coli* cultured in control conditions. This allowed the screening of changes in the levels of tRNAs responsible for the decoding of 10 different amino acids during translation. Out of 10 tested tRNAs, only tRNA^Gly^ showed a statistically significant decrease in the levels of active tRNA after stress by exposure to either paraquat or H_2_O_2_ (Figure 1A). Previous reports have shown a general decrease in total tRNA levels under oxidative stress in minimal media (Zhong et al., 2015; Zhu and Dai, 2019). In contrast, we observed that tRNA levels remained fairly constant (Figure 1B), suggesting the observed decrease in the levels of active tRNA^Gly^ was a specific response to oxidative stress. Since we observe comparable effects on tRNA^Gly^ after addition of either H_2_O_2_ or paraquat, we confined additional studies to the effects of paraquat, that is continually reduced by the cellular NADPH pool (Hassan and Fridovich, 1979) producing a constant, readily reproducible oxidative stress. Furthermore, as oxidative stress is known to inactivate dihydroxyacid dehydratase and consequentially impair the synthesis of branched amino acids (Imlay, 2008), we added Leu, Val, and Ile to cultures when using minimal media.

### The Effect of Oxidative Stress on Specific tRNA^Gly^ Isoacceptors

*E. coli* has six-genes coding for tRNA^Gly^. Four of them (*glyV*, *glyW*, *glyX*, and *glyY*) code for identical tRNAs that are indistinguishable by Northern blot. These tRNAs have a GCC anticodon (tRNA^Gly^_GCC_) that decodes GGC and GGU, two codons that are used at high frequency in *E. coli*. A fifth gene, *glyU*, codes for tRNA^Gly^_CCC_ that exclusively decodes GGG codons. A sixth gene, *glyT*, codes for tRNA^Gly^_UCC_ that decodes GGA and GGG codons. Codons decoded by tRNA^Gly^_CCC_ and tRNA^Gly^_UCC_ are used with a lower frequency (Table 1 and Supplementary Figure S2). Genes *glyV*, *glyX*, and *glyY* are clustered together in a putative operon, while the other genes for tRNA^Gly^ are either not clustered (*glyU*) or clustered with genes for other tRNAs (*glyT* and *glyW*; Supplementary Figure S3). We tested whether changes in the expression levels of specific tRNA^Gly^ isoacceptors correlate with the observed differences in active tRNA levels during oxidative stress. Northern blot analyses indicated that levels of all tRNA^Gly^ isoacceptors were unaltered under oxidative stress (Figure 1C). The lack of variation in tRNA^Gly^ isoacceptor expression suggested that the changes in active tRNA^Gly^ levels might instead result from chemical modifications that impair their interaction with GlyRS during aminoacylation. We determined *in vivo* aminoacylation levels for tRNA^Gly^ directly by subjecting total tRNA pools to periodate oxidation and β-elimination followed by Northern blot (Salazar et al., 2004). Through this treatment, deaminoacylated tRNAs loses their terminal adenine and migrate faster in polyacrylamide electrophoresis gels while aa-tRNAs are protected by the amino acid and retain their original length and electrophoretic mobility. We observed decreases in the *in vivo* aminoacylation levels of the three tRNA^Gly^ isoacceptors under oxidative stress. While ~60−80% of each tRNA^Gly^ isoacceptor is aminoacylated under control conditions, after paraquat addition these levels decreased to around ~30−50% (Figure 1D). However, a stronger decrease was observed for tRNA^Gly^_UCC_ (from ~60 to ~30%) and tRNA^Gly^_CCC_ (from ~80 to ~30%) that are less abundant than tRNA^Gly^_GCC_ (from ~70 to ~50%). In similar experiments, we found that oxidative stress induced by paraquat produces only minor alterations to the concentration and aminoacylation levels of other tRNAs such as tRNA^Trp^, tRNA^Try^, and initiator tRNA^fMet^, confirming that the observed deaminoacylation is a specific behavior of tRNA^Gly^ (Supplementary Figure S4). Next, we hypothesized that tRNA^Gly^ inactivation might be mediated through differences in the levels of chemical modification of the tRNA^Gly^ isoacceptors. Individual tRNA^Gly^ isoacceptors were purified and analyzed by quantitative RNA mass spectrometry. Surprisingly, while we detected all modified nucleosides that are known for each isoacceptor, we did not observe quantitative differences between the stress and the control samples (data not shown). However, these results do not necessarily invalidate our hypothesis, as abasic sites and unknown oxidation products of modified tRNA^Gly^ nucleotides that might inactivate the tRNAs could have escaped our analysis. Finally, modifications might have been lost as a result of heating in the presence of a reducer during purification of individual tRNA^Gly^ isoacceptors.

**Table 1:** tRNA^Gly^ coded in *E. coli* K-12 MG1655 genome.

tRNA^Gly^ genes	Anticodon	Decoded codon(s) (usage frequency^*^)	% of total tRNA^**^
*glyV*, *glyW*, *glyX*, *glyY*	GCC	GGC (2.96%)	6.76%
		GGU (2.47%)					
*glyU*	CCC	GGG (1.11%)	3.31%^***^			
*glyT*	UCC	GGA (0.79%)	
		GGG (1.11%)					
-	ACC	-	

* Based on data available at GtRNAdb (Chan and Lowe, 2009).

** As estimated in exponentially growing cells (Dong et al., 1996).

*** Spots of tRNA^Gly^_CCC_ and tRNA^Gly^_UCC_ were not resolved, so the authors reported the total abundance for both tRNA isoacceptor together.

### Effect of Oxidative Stress on Translation of Gly Codons

**Figure 2:**
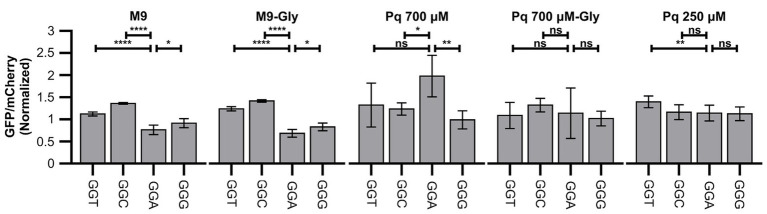
Oxidative stress alters translation of Gly codons. Figure shows green fluorescent protein (GFP) fluorescence normalized by fluorescence of mCherry in diverse strains and conditions. Data were additionally normalized dividing by the GFP/mCherry ratio of the control strain (reporter S1, without additional codons). Four identical Gly codons were cloned in fusion to GFP and fluorescence measured in control media (M9), control media with Gly (M9-Gly), media with high concentration of paraquat in the absence (PQ 700 μM) or presence (PQ 700 μM-Gly), or media with low concentration of paraquat (PQ 250 μM). ^****^p ≤ 0.0001, ^**^p ≤ 0.01, ^*^p < 0.05, one way ANOVA with Dunnett versus GGA strain for each condition (*n* ≥ 3).

Changes in aa-tRNA concentration are likely to alter the translation efficiency of the codons they decode. As each tRNA^Gly^ isoacceptor decodes a different set of codons, this could potentially lead to codon-dependent changes in gene expression, which in turn could give rise to distinct phenotypes. To determine whether the observed decrease in active tRNA^Gly^ affects the decoding efficiency of the corresponding Gly codons, we generated reporter constructs where we fused GFP to four contiguous identical Gly codons using mCherry as an internal control of transcript levels and global alterations of translation initiation. We used these reporters to test whether GFP production was affected in response to oxidative stress conditions where we observed changes in active tRNA^Gly^ levels and aminoacylation (Figure 1). We previously used this strategy to analyze the role of translation elongation factor P during the translation of several codon patterns (Elgamal et al., 2014) and the effect of natural changes in tRNA gene copy numbers on codon translation (Rojas et al., 2018). We first tested GFP production in M9 minimal medium containing glycerol and branched amino acids, both in the presence and absence of Gly. In both conditions, we observed a higher GFP/mCherry ratio when using reporters containing the frequent GGC or GGU codons as compared to the infrequently used codons (GGA or GGG). This shows that the method is sufficiently sensitive to differentiate between the translation efficiency of different Gly codons [Figure 2; ratio between GFP/mCherry values for the most frequently used codon (GGC) and least frequently used codon (GGA) is ~1.75 fold in absence of Gly]. Additionally, this suggests that translation of four contiguous Gly codons has a similar or slower speed than initiation that is usually considered the limiting step of translation (Subramaniam et al., 2013b; Hersch et al., 2014). Otherwise, translation of the four reporters would have produced similar amounts of GFP. We then repeated the experiment in a strain where two out of four of the genes coding for tRNA^Gly^_GCC_ (*glyVX*) were deleted, leading to a decrease in the levels of the tRNA^Gly^_GCC_ (data not shown). Decreasing the levels of tRNA^Gly^_GCC_ induced a lower translation of the *gfp* genes enriched in GGT or GGC codons (that are directly translated by tRNA^Gly^_GCC_) and only minor effects on *gfp* enriched for the two other Gly codons, indicating that the method is sensitive to changes in tRNA levels (Supplementary Figure S5).

While adding paraquat to minimal media at the time of reporter induction completely stopped cell replication, GFP and mCherry synthesis continued (although at much lower levels) indicating that cells can still transcribe and translate their genes following initiation of oxidative stress. The addition of paraquat induced a strong decrease in the production of both GFP and mCherry in the presence or absence of Gly (around 10 and 15 fold decrease for GFP/OD_600_ and mCherry/OD_600_ values, respectively, for the strain carrying the control plasmid; data not shown). When oxidative stress was induced in the presence of Gly, no difference was observed between the translation of each Gly codon (Figure 2). This result suggests that the decrease in the rate of translation initiation or another limiting step is much stronger than any effect on Gly codons translation, making differences in Gly translation unmeasurable. Instead, when Gly was absent from the culture media, differences between translation of each Gly codon were readily measurable and we observed that, unexpectedly, translation of GGA shifted from being the slower Gly codon to being the fastest codon (Figure 2). This suggests that under this condition, not only a fraction of tRNA^Gly^ isotypes are inactivated, but additionally, Gly becomes limiting. Thus, a reduced aminoacylation derived from low Gly availability (Böck and Neidhardt, 1966; Folk and Berg, 1970a,b; Subramaniam et al., 2013b) plus tRNA^Gly^ inactivation made Gly codon translation slow enough to produce measurable differences in GFP production. In agreement with this interpretation, if we reduce the concentration of paraquat added to the media lacking Gly from 700 to 250 μM, presumably decreasing the degree of tRNA^Gly^ inactivation, the differences between Gly codons are also not observed (Figure 2). Although less likely, an alternative interpretation that we cannot rule out is that addition of Gly somehow protects tRNA^Gly^ from inactivation without producing a similar degree of protection to translation initiation. Following this interpretation, under reduced paraquat concentrations, the fraction of inactivated tRNA^Gly^ would be much smaller than inhibition of translation initiation.

### Effect of Oxidative Stress on Translation of GGA Codons in Natural Context

**Figure 3:**
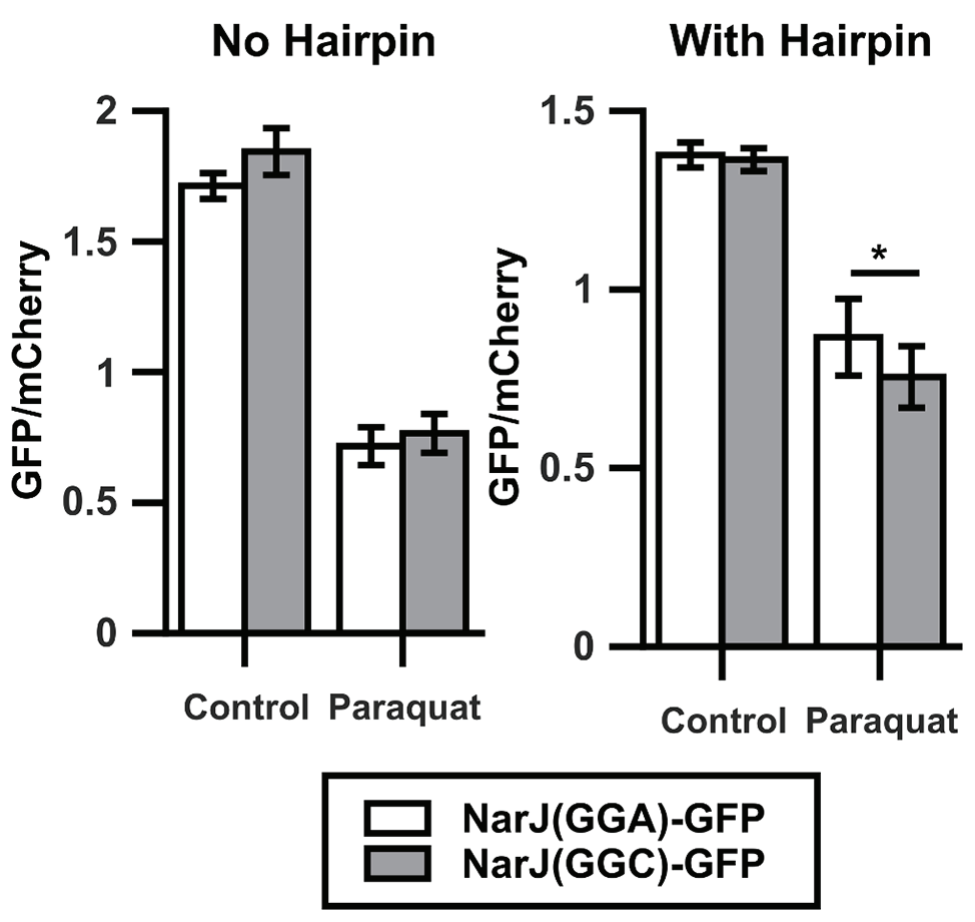
Oxidative stress alters translation of Gly codons in natural context. Left graph shows the GFP/mCherry fluorescence ratio for *narJ* [NarJ(GGA)-GFP, white] or a mutant of the gene where a contiguous GGA pair was changed for a pair of GGC [NarJ(GGC)-GFP, gray] cloned in fusion to GFP. Shown in the right graph are similar experiments performed after introducing a hairpin that prevents ribosome sliding between the sequences coding for GFP and mCherry. ^*^p < 0.05, two-tailed *t*-test (*n* = 3).

The results shown here indicate that oxidative stress caused by 700 μM paraquat alters translation of Gly codons when located in the context of four continued identical Gly codons within the *gfp* gene. These alterations are stronger for the most frequently used codons, transforming the most infrequently used GGA codon into the fastest Gly codon under strong oxidative stress. Nevertheless, in *E. coli* K-12 MG1655, GGA is never found in the context of four consecutive identical codons, questioning the relevance of our observation in natural genes. To verify the effect of paraquat on translation of Gly codons located in their natural context, we cloned WT *narJ* (coding for nitrate reductase molybdenum cofactor assembly chaperon) and a version where its two contiguous GGA codons were mutated to GGC in a translational fusion to *gfp* to form the *narJ(GGA)-gfp* and *narJ(GGC)-gfp* genes. Like in the previous experiments, we used mCherry as an internal control. We did not observe a significant difference between changes in GFP/mCherry fluorescence ratios under control and stress conditions for the *narJ(GGA)-gfp* and *narJ(GGC)-gfp* strains (Figure 3, left panel). The sensitivity of our reporter may be decreased, if ribosomes that have translated *gfp* slide and initiate *mCherry* translation (70S-scanning initiation; Yamamoto et al., 2016), as then *mCherry* translation would not be completely independent of *gfp* translation. To avoid the possible effects of 70S-scanning, we introduced a sequence which is predicted to form a stable hairpin between both genes and has been previously shown to prevent ribosome sliding (Yamamoto et al., 2016). Using this construct, we observe a ~20% higher GFP/mCherry fluorescence ratio under oxidative stress when *narJ* is coded using GGA codons than when using GGC codons (Figure 3, right panel). This indicates that although under oxidative stress, GGA translation is less inhibited than translation of other Gly codons, and its effect on the amount of protein produced will strongly depend on the context where the codon is located.

### tRNA^Gly^ Modulates the Response to Oxidative Stress

**Figure 4:**
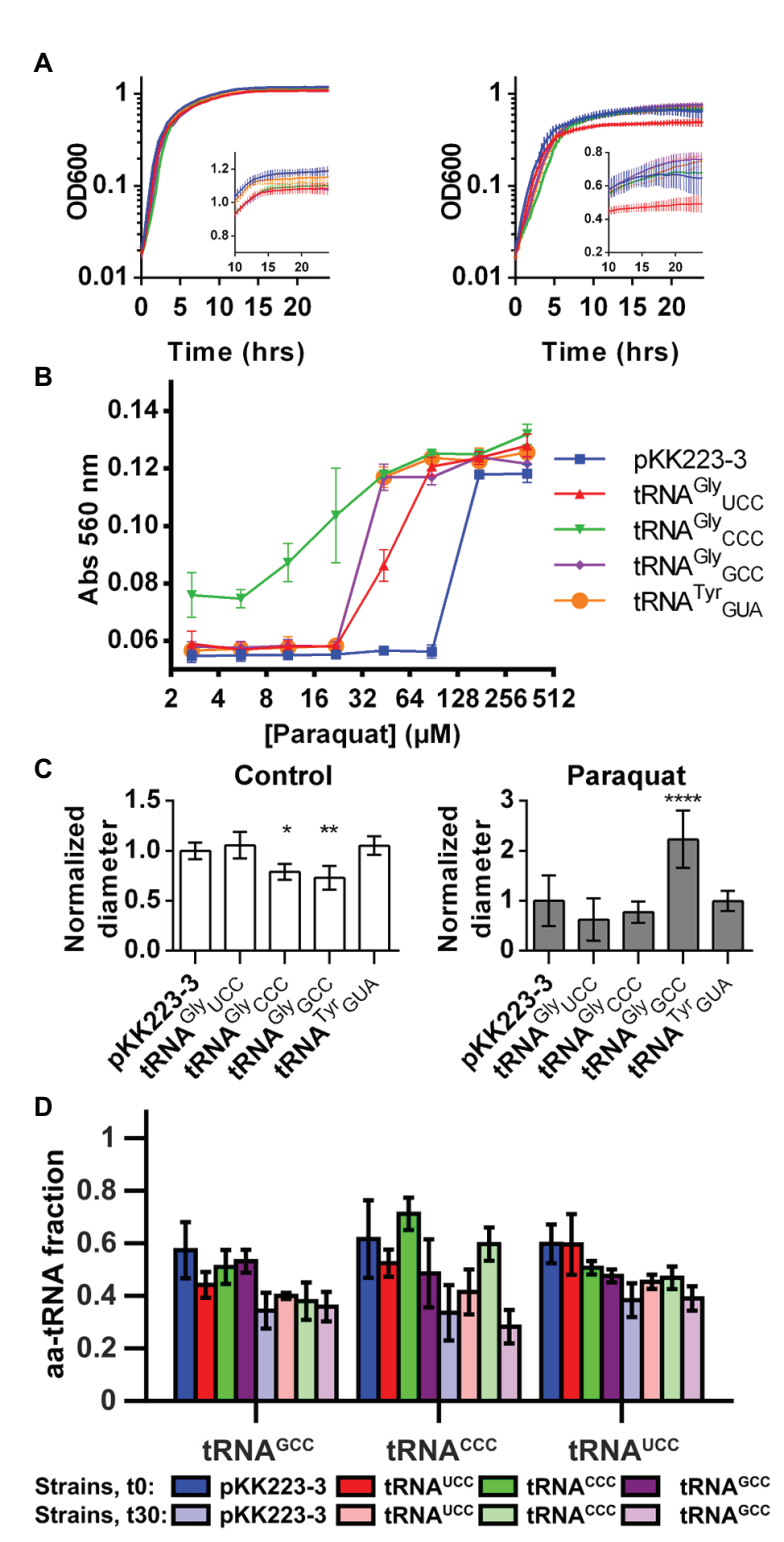
Overproduction of tRNA^Gly^ alters the response of *E. coli* to oxidative stress. The effect of overproduction of diverse tRNA^Gly^ isoacceptors on the response to oxidative stress induced by paraquat was studied. **(A)** Effect of tRNA^Gly^ overproduction over growth curves performed at 37°C in control media (left) or media with paraquat (stress condition, right). Inset graph shows same data in linear scale for time points above 10 h. tRNA^Gly^_CCC_: green, tRNA^Gly^_GCC_: purple, tRNA^Gly^_UCC_: red, tRNA^Tyr^_GUA_: orange, and empty pKK223-3 plasmid: blue (*n* = 8). **(B)** Effect of tRNA^Gly^ overproduction on the fermentation of lactose as measured by changes in media pH after incubation at diverse paraquat concentrations. Higher absorbance indicates higher pH (lower fermentation of the carbohydrate). tRNA^Gly^_CCC_: green inverted triangles, tRNA^Gly^_GCC_: purple rhombus, tRNA^Gly^_UCC_: red triangles, tRNA^Tyr^_GUA_: orange circles, and empty pKK223-3 plasmid: blue squares (*n* = 3). **(C)** Effect of tRNA^Gly^ overproduction on bacterial motility as measured by changes in the diameter of colonies cultured on low agar LB plates in the absence (left) or presence (right) of paraquat. Data in graph represent the diameter of colonies normalized dividing by the average diameter of the control colonies. Each bar FIGURE 4represents the average of at least four replicates. ^****^p ≤ 0.0001, ^**^p ≤ 0.01, ^*^p ≤ 0.05, one way ANOVA with Dunnett versus strain with empty plasmid at the corresponding condition. **(D)** Fraction of tRNA^Gly^ isoacceptors that is aminoacylated in strains overproducing diverse tRNA^Gly^ isoacceptors. Color represents the tRNA isoacceptor that is overproduced in strains where aminoacylation was quantified. tRNA^Gly^_CCC_: green, tRNA^Gly^_GCC_: purple, tRNA^Gly^_UCC_: red, empty pKK223-3 plasmid: blue. Darker colors correspond to measurements performed before paraquat addition, while light colors represent aminoacylation 30 min after addition of the stressor. Each bar represents the average of at least three replicates.

Alterations of translation efficiency of Gly codons probably alter level and/or cotranslational folding of several proteins under oxidative stress. In order to confirm that alterations in the active tRNA^Gly^ pool may affect the response to oxidative stress, we studied the response to oxidative stress in strains overproducing each tRNA^Gly^ isoacceptor from an IPTG inducible plasmid (pKK223-3). In all these experiments, the empty plasmid was used as a control for non-specific effects of the plasmid, and a plasmid coding for a tRNA that did not show changes in our aminoacylation experiments (tRNA^Tyr^_GUA_) was used as a control for the non-specific effects of tRNA overproduction. Overproduction of any of the tested tRNAs (including tRNA^Tyr^_GUA_) increased the sensitivity of carbohydrate fermentation to paraquat as measured by changes in the pH of culture media. Nevertheless, the effect was much stronger for the strain producing tRNA^Gly^_CCC_. Similarly, all the other tested phenotypes were also dependent on the overproduction of individual isoacceptors. For instance, while the stronger effect of overproduction of tRNA^Gly^_GCC_ was in preventing the loss of bacterial motility (measured as changes in colony diameter in low agar LB plates), overproduction of tRNA^Gly^_UCC_ mostly reduced culture yield (measured in 96-well plate cultures). As mentioned above, overproduction of tRNA^Gly^_CCC_ mainly increased the sensitivity of carbohydrate fermentation to paraquat (Figure 4 and Supplementary Figure S6). In contrast to these phenotypes, cells overproducing these tRNAs did not show any change in their respective MIC for paraquat (25 μM in M9 media supplemented with branched amino acids).

In addition to generating idiosyncratic phenotypes, overproduction of each tRNA^Gly^ induced distinct changes in the aminoacylation of the other tRNA^Gly^ isoacceptors (Figure 4D). For instance, overproduction of tRNA^Gly^_GCC_ led to a ~10% decrease of the basal levels of aminoacylation of all tRNA^Gly^ isoacceptors. In contrast, tRNA^Gly^_UCC_ overproduction only altered basal aminoacylation of the other tRNA^Gly^ isoacceptors, but not its own. Finally, overproduction of tRNA^Gly^_CCC_ increased its basal level of aminoacylation while inducing a decrease in aminoacylation of other tRNA^Gly^ isoacceptors. Under oxidative stress, the effects were similarly diverse, with tRNA^Gly^_GCC_ inducing a decrease in tRNA^Gly^_CCC_ aminoacylation but tRNA^Gly^_UCC_ and, in particular, tRNA^Gly^_CCC_ inducing an increase in tRNA^Gly^_CCC_ aminoacylation level. Overall, under oxidative stress, the aminoacylation of the most abundant tRNA^Gly^ isoacceptor (tRNA^Gly^_GCC_) was the least sensitive to the levels of other tRNA^Gly^ isoacceptors, while tRNA^Gly^_CCC_ showed the highest sensitivity.

In summary, the specificity of the phenotypes induced by overproduction of each tRNA^Gly^ isoacceptor and the fact that most were observed under oxidative stress but not during normal growth suggests that these effects are triggered by individual tRNA^Gly^ isoacceptors and are not simply secondary effects of tRNA overproduction. Nevertheless, it is currently not possible to determine a cause-effect relation between particular tRNAs and phenotypes because changes in the levels of any tRNA^Gly^ isoacceptor alter the aminoacylation of other tRNAs.

## Discussion

### Regulation of Translation by Changes in tRNA Concentration

Here, we demonstrate that oxidative stress induces specific alterations in the tRNA^Gly^ pools and concurrent changes in Gly codon translation rates. Nevertheless, changes in protein levels are only observed under strong oxidative stress, low Gly availability, and particular genetic context. Taken together, our findings suggest that diversity of codon translation speeds is only observed when the speed of Gly codon translation is similar to or slower than translation initiation which is usually considered the limiting step of the complete process (Hersch et al., 2014; Subramaniam et al., 2014). Thus, it appears that the translational response to oxidative stress is complex and effects on elongation are only observed under the most hostile conditions. Either lower paraquat concentration or higher Gly availability induces a condition where changes in *gfp* codons do not affect protein production.

In agreement with our observations, reports of experiments performed in other *E. coli* strains also indicate that the relevance of elongation in determining the overall speed of protein production increases at stronger oxidative stress conditions, in this case induced by higher concentrations of H_2_O_2_ (Zhu and Dai, 2019). In contrast to what we have observed, but in agreement with previous reports (Zhong et al., 2015), the authors of these experiments observe a decrease in the concentration of all tRNAs. Thus, the translational response to oxidative stress seems to be strain dependent. It is currently difficult to predict what determines these different behaviors between *E. coli* strains. For example, the lack of a toxin-antitoxin system or a smaller nuclease activity in the strain we used could prevent tRNA cleavage under oxidative stress. Alternatively, a decreased protease activity or increased amino acid usage might produce stronger limitations of amino acid availability in strains used by other groups, thereby inducing degradation of tRNAs (Svenningsen et al., 2017).

Further research will be required to clarify the peculiarities that induce different behaviors between *E. coli* strains. Nevertheless, our data show that in *E. coli* K-12 MG1655, an important model strain, only tRNA^Gly^ is inactivated under oxidative stress. Although all isoacceptors are deacylated, the decrease in the speed of GGA translation is smaller than observed for other Gly codons. This should allow preferential translation of GGA enriched genes under oxidative stress. As mentioned previously, GGA is used with a lower frequency than other Gly codons in *E. coli* K-12 MG1655 (Table 1; Supplementary Figure S2; Supplementary Table S1). Only 79 genes (~1.8% of all *E. coli* K-12 MG1655 genes) use GGA as 3% or more of their codons. Many of the genes that use GGA codons with high frequency are implicated in the negative regulation of cell growth and cellular defense, including toxins from three toxin-antitoxin systems (*chpB*, *mazF*, and *ralR*) that might explain the differences observed in growth after overproduction of tRNA^Gly^_UCC_. The high abundance of GGA codons in genes such as *rmf*, which is involved in ribosome hibernation during stationary phase or *yciH* that binds the ribosome and alters the expression of stress response genes, and growth in minimal medium could further explain some of the observed growth phenotypes. Also, the enrichment of genes implicated in cell adhesion or motility (*yraK*, *ydeQ*, *yadK*, *flhE*, and *chaC*) could explain different motility behaviors between strains overexpressing the different tRNA^Gly^ genes. A comprehensive list of functions enriched in the list of genes with high GGA codon usage is given in Supplementary Table S2. Nevertheless, care should be taken when extrapolating results obtained by using fluorescent reporters to these GGA-rich genes, as our results indicate that sensitivity to oxidative stress may strongly depend on the context where GGA codons are located. This context sensitivity may derive from different speeds of translation depending on neighboring codons (Chevance et al., 2014), for instance, due to interactions with other tRNAs in the ribosome (Buchan et al., 2006). As we are analyzing translation of full-length genes and not isolated GGA codons, different sensitivities may additionally arise from a slow translation initiation or specific patterns of amino acids or codons where translation elongation is very slow, making any change in GGA translation undetectable due to other limiting steps in translation (Hersch et al., 2014). As mentioned previously, this might explain the lack of differences between translation of each Gly codon under some conditions. Oxidation of ribosomal RNA (rRNA) or proteins (Katz and Orellana, 2012; Liu et al., 2012; Willi et al., 2018) might explain such a decrease in translation initiation. In this context, the fact that the anti-Shine-Dalgarno of *E. coli* presents repeats (ACCUCC) of the tRNA^Gly^ anticodon sequences (NCC) might suggest a similar modification mechanism. Nevertheless, the little available data (Willi et al., 2018) suggests that the 3' extreme is more resistant to oxidative stress derived modifications than other segments of the rRNA.

Based on current data, it is difficult to determine why translation of GGA is less inhibited than translation of the other Gly codons. One possibility is that there is a lower requirement of aa-tRNA^Gly^_UCC_ as a consequence of the low frequency of usage of GGA codons (Table 1; Supplementary Figure S2). If true, this would limit the sensitivity of translation to changes in the levels of active tRNA^Gly^_UCC_. Nevertheless, relations between tRNA and translation appear to be complex, and the effect of changes of a single tRNA might be difficult to predict. For instance, while we observe that increasing the concentration of tRNA^Gly^_GCC_ may result in significant alterations in the levels of aminoacylation of the other tRNA^Gly^ isoacceptors (Figure 4D), we also found that at least under some conditions, a decrease in concentration of the same tRNA can have very limited effects on translation of the codons not directly translated by the affected tRNA (Supplementary Figure S5).

### Final Remarks

Recent research on the effects of oxidative stress on the bacterial translation machinery has shown a very diverse set of effects ranging from changes in error rates (Ling and Söll, 2010; Wu et al., 2014; Steiner et al., 2019) to translation inhibition (Kojima et al., 2009; Nagano et al., 2015; Zhong et al., 2015; Yutthanasirikul et al., 2016; Zhu and Dai, 2019). Such results appear to derive from alterations to all components of the machinery such as changes in tRNA (Katz and Orellana, 2012; Liu et al., 2012; Zhong et al., 2015; Zhu and Dai, 2019), aminoacyl-tRNA synthetases (Ling and Söll, 2010; Katz and Orellana, 2012; Wu et al., 2014; Steiner et al., 2019), ribosomes (Katz and Orellana, 2012; Liu et al., 2012; Willi et al., 2018), and elongation factors (Kojima et al., 2009; Katz and Orellana, 2012; Nagano et al., 2015; Yutthanasirikul et al., 2016). Nevertheless, comparison of our results with these previous reports indicates that the relevance of each of these changes to bacterial adaptation depends not only on the culture conditions but also on the strains being analyzed. Thus, further studies will be required to understand the relevance of alterations in each component of the translation machinery in the adaptation to diverse degrees of stress and how the genetic background of each strain determines this response.

## Data Availability Statement

All datasets generated for this study are included in the article/Supplementary Material.

## Author Contributions

Aminoacylation experiments were performed by AK and LL. tRNA level quantifications were performed by AP and LL. Phenotypic characterization was performed by LL and AP. tRNA purification was performed by VB, mass spectrometry experiments by SK, and mass spectrometry data analyses by SK and JL. Cloning, mutagenesis, and GFP/mCherry analyses were performed by SE, DG, and LL. Codon enrichment analysis was performed by AK. MI, AK, and SL supervised experiments and interpreted the data. AK wrote the paper with contributions from all authors. All authors contributed to the article and approved the submitted version.

### Conflict of Interest

The authors declare that the research was conducted in the absence of any commercial or financial relationships that could be construed as a potential conflict of interest.

## References

[ref1] Alings F. Sarin L. P. Fufezan C. Drexler H. C. A. Leidel S. A. (2015). An evolutionary approach uncovers a diverse response of TRNA 2-thiolation to elevated temperatures in yeast. RNA 21, 202–212. 10.1261/rna.048199.114, PMID: 25505025 PMC4338348

[ref2] Bald T. Barth J. Niehues A. Specht M. Hippler M. Fufezan C. (2012). pymzML--Python module for high-throughput bioinformatics on mass spectrometry data. Bioinformatics 28, 1052–1053. 10.1093/bioinformatics/bts066, PMID: 22302572

[ref3] Blattner F. R. Plunkett G. Bloch C. A. Perna N. T. Burland V. Riley M.. (1997). The complete genome sequence of *Escherichia coli* K-12. Science 277, 1453–1462. 10.1126/science.277.5331.1453, PMID: 9278503

[ref4] Blondel C. J. Jiménez J. C. Leiva L. E. Alvarez S. A. Pinto B. I. Contreras F.. (2013). The type VI secretion system encoded in *Salmonella* pathogenicity island 19 is required for *Salmonella enterica* serotype gallinarum survival within infected macrophages. Infect. Immun. 81, 1207–1220. 10.1128/IAI.01165-12, PMID: 23357385 PMC3639620

[ref5] Boccaletto P. Machnicka M. A. Purta E. Piatkowski P. Baginski B. Wirecki T. K.. (2017). MODOMICS: a database of RNA modification pathways. 2017 update. Nucleic Acids Res. 46, D303–D307. 10.1093/nar/gkx1030, PMID: 29106616 PMC5753262

[ref6] Böck A. Neidhardt F. C. (1966). Location of the structural gene for Glycyl ribonucleic acid synthetase by means of a strain of *Escherichia coli* possessing an unusual enzyme. Z. Vererbungsl. 98, 187–192. 10.1007/BF00888946, PMID: 4863694

[ref7] Buchan J. R. Aucott L. S. Stansfield I. (2006). tRNA properties help shape codon pair preferences in open reading frames. Nucleic Acids Res. 34, 1015–1027. 10.1093/nar/gkj488, PMID: 16473853 PMC1363775

[ref8] Chambers M. C. Maclean B. Burke B. Amodei D. Ruderman D. L. Neumann S.. (2012). A cross-platform toolkit for mass spectrometry and proteomics. Nat. Biotechnol. 30, 918–920. 10.1038/nbt.2377, PMID: 23051804 PMC3471674

[ref9] Chan P. P. Lowe T. M. (2009). GtRNAdb: a database of transfer RNA genes detected in genomic sequence. Nucleic Acids Res. 37(Suppl. 1), D93–D97. 10.1093/nar/gkn787, PMID: 18984615 PMC2686519

[ref10] Charbon G. Bjørn L. Mendoza-Chamizo B. Frimodt-Møller J. Løbner-Olesen A. (2014). Oxidative DNA damage is instrumental in hyperreplication stress-induced inviability of *Escherichia coli*. Nucleic Acids Res. 42, 13228–13241. 10.1093/nar/gku1149, PMID: 25389264 PMC4245963

[ref11] Chevance F. F. V. Le Guyon S. Hughes K. T. (2014). The effects of codon context on in vivo translation speed. PLoS Genet. 10:e1004392. 10.1371/journal.pgen.1004392, PMID: 24901308 PMC4046918

[ref12] Chiang S. M. Schellhorn H. E. (2012). Regulators of oxidative stress response genes in *Escherichia coli* and their functional conservation in bacteria. Arch. Biochem. Biophys. 525, 161–169. 10.1016/j.abb.2012.02.007, PMID: 22381957

[ref13] Cohen N. R. Lobritz M. A. Collins J. L. (2013). Microbial persistence and the road to drug resistance. Cell Host Microbe 13, 632–642. 10.1016/j.chom.2013.05.009, PMID: 23768488 PMC3695397

[ref14] Datsenko K. A. Wanner B. L. (2000). One-step inactivation of chromosomal genes in *Escherichia coli* K-12 using PCR products. Proc. Natl. Acad. Sci. U. S. A. 97, 6640–6645. 10.1073/pnas.120163297, PMID: 10829079 PMC18686

[ref15] Dong H. Nilsson L. Kurland C. G. (1996). Co-variation of tRNA abundance and codon usage in *Escherichia coli* at different growth rates. J. Mol. Biol. 260, 649–663. 10.1006/jmbi.1996.0428, PMID: 8709146

[ref16] Elgamal S. Katz A. Hersch S. J. Newsom D. White P. Navarre W. W.. (2014). EF-P dependent pauses integrate proximal and distal signals during translation. PLoS Genet. 10:e1004553. 10.1371/journal.pgen.1004553, PMID: 25144653 PMC4140641

[ref17] Folk W. R. Berg P. (1970a). Characterization of altered forms of Glycyl transfer ribonucleic acid synthetase and the effects of such alterations on aminoacyl transfer ribonucleic acid synthesis in vivo. J. Bacteriol. 102, 204–212. 4908672 10.1128/jb.102.1.204-212.1970 PMC284987

[ref18] Folk W. R. Berg P. (1970b). Isolation and partial characterization of *Escherichia coli* mutants with altered Glycyl transfer ribonucleic acid synthetases. J. Bacteriol. 102, 193–203. 4908671 10.1128/jb.102.1.193-203.1970 PMC284986

[ref19] Gambino M. Cappitelli F. (2016). Mini-review: biofilm responses to oxidative stress. Biofouling 32, 167–178. 10.1080/08927014.2015.1134515, PMID: 26901587

[ref20] Ha D. Kuchma S. L. O’Toole G. A. (2014). Plate-based assay for swimming motility in *Pseudomonas aeruginosa*. Methods Mol. Biol. 1149, 59–65. 10.1007/978-1-4939-0473-0_7, PMID: 24818897 PMC9007281

[ref21] Hassan H. M. Fridovich I. (1979). Paraquat and *Escherichia coli*. Mechanism of production of extracellular superoxide radical. J. Biol. Chem. 254, 10846–10852. PMID: 227855

[ref22] Henriques M. X. Catalão M. J. Figueiredo J. Gomes J. P. Filipe S. R. (2013). Construction of improved tools for protein localization studies in *Streptococcus pneumoniae*. PLoS One 8:e55049. 10.1371/journal.pone.0055049, PMID: 23349996 PMC3551898

[ref23] Hersch S. J. Elgamal S. Katz A. Ibba M. Navarre W. W. (2014). Translation initiation rate determines the impact of ribosome stalling on bacterial protein synthesis. J. Biol. Chem. 289, 28160–28171. 10.1074/jbc.M114.593277, PMID: 25148683 PMC4192472

[ref24] Imlay J. A. (2008). Cellular defenses against superoxide and hydrogen peroxide. Annu. Rev. Biochem. 77, 755–776. 10.1146/annurev.biochem.77.061606.161055, PMID: 18173371 PMC3057177

[ref25] Imlay J. A. (2013). The molecular mechanisms and physiological consequences of oxidative stress: lessons from a model bacterium. Nat. Rev. Microbiol. 11, 443–454. 10.1038/nrmicro3032, PMID: 23712352 PMC4018742

[ref26] Katz A. Elgamal S. Rajkovic A. Ibba M. (2016). Non-canonical roles of tRNAs and tRNA mimics in bacterial cell biology. Mol. Microbiol. 101, 545–558. 10.1111/mmi.13419, PMID: 27169680 PMC5003029

[ref27] Katz A. Orellana O. (2012). “Protein synthesis and the stress response” in Cell-free protein synthesis. ed. Biyani M. (Rijeka, Croatia: IntechOpen), 111–134.

[ref28] Keseler I. M. Mackie A. Santos-Zavaleta A. Billington R. Bonavides-Martínez C. Caspi R.. (2017). The EcoCyc database: reflecting new knowledge about *Escherichia coli* K-12. Nucleic Acids Res. 45, D543–D550. 10.1093/nar/gkw1003, PMID: 27899573 PMC5210515

[ref29] Kojima K. Motohashi K. Morota T. Oshita M. Hisabori T. Hayashi H.. (2009). Regulation of translation by the redox state of elongation factor G in the cyanobacterium *Synechocystis* sp. PCC 6803. J. Biol. Chem. 284, 18685–18691. 10.1074/jbc.M109.015131, PMID: 19447882 PMC2707220

[ref30] Leufken J. Niehues A. Sarin L. P. Wessel F. Hippler M. Leidel S. A.. (2017). pyQms enables universal and accurate quantification of mass spectrometry data. Mol. Cell. Proteomics 16, 1736–1745. 10.1074/mcp.M117.068007, PMID: 28729385 PMC5629261

[ref31] Lewis K. (2010). Persister cells. Annu. Rev. Microbiol. 64, 357–372. 10.1146/annurev.micro.112408.134306, PMID: 20528688

[ref32] Ling J. Söll D. (2010). Severe oxidative stress induces protein mistranslation through impairment of an aminoacyl-tRNA synthetase editing site. Proc. Natl. Acad. Sci. U. S. A. 107, 4028–4033. 10.1073/pnas.1000315107, PMID: 20160114 PMC2840151

[ref33] Liu M. Gong X. Alluri R. K. Wu J. Sablo T. Li Z. (2012). Characterization of RNA damage under oxidative stress in *Escherichia coli*. Biol. Chem. 393, 123–132. 10.1515/hsz-2011-0247, PMID: 22718628 PMC3404489

[ref34] Martens L. Chambers M. Sturm M. Kessner D. Levander F. Shofstahl J.. (2011). mzML--a community standard for mass spectrometry data. Mol. Cell. Proteomics 10:R110.000133. 10.1074/mcp.R110.000133, PMID: 20716697 PMC3013463

[ref35] Nagano T. Yutthanasirikul R. Hihara Y. Hisabori T. Kanamori T. Takeuchi N.. (2015). Oxidation of translation factor EF-G transiently retards the translational elongation cycle in *Escherichia coli*. J. Biochem. 158, 165–172. 10.1093/jb/mvv026, PMID: 25742739

[ref36] Nguyen G. T. Green E. R. Mecsas J. (2017). Neutrophils to the ROScue: mechanisms of NADPH oxidase activation and bacterial resistance. Front. Cell. Infect. Microbiol. 7:373. 10.3389/fcimb.2017.00373, PMID: 28890882 PMC5574878

[ref37] Raczniak G. Becker H. D. Min B. Söll D. (2001). A single amidotransferase forms asparaginyl-tRNA and glutaminyl-tRNA in *Chlamydia trachomatis*. J. Biol. Chem. 276, 45862–45867. 10.1074/jbc.M109494200, PMID: 11585842

[ref38] Rojas J. Castillo G. Leiva L. E. Elgamal S. Orellana O. Ibba M.. (2018). Codon usage revisited: lack of correlation between codon usage and the number of tRNA genes in enterobacteria. Biochem. Biophys. Res. Commun. 502, 450–455. 10.1016/j.bbrc.2018.05.168, PMID: 29859934 PMC6024254

[ref39] Rui B. Shen T. Zhou H. Liu J. Chen J. Pan X.. (2010). A systematic investigation of *Escherichia coli* central carbon metabolism in response to superoxide stress. BMC Syst. Biol. 4:122. 10.1186/1752-0509-4-122, PMID: 20809933 PMC2944137

[ref40] Salazar J. C. Ambrogelly A. Crain P. McCloskey J. A. Söll D. (2004). A truncated aminoacyl-tRNA synthetase modifies RNA. Proc. Natl. Acad. Sci. U. S. A. 101, 7536–7541. 10.1073/pnas.0401982101, PMID: 15096612 PMC419641

[ref41] Sarin L. P. Kienast S. D. Leufken J. Ross R. Dziergowska A. Debiec K.. (2018). Nano LC-MS using capillary columns enables accurate quantification of modified ribonucleosides at low femtomol levels. RNA 24, 1403–1417. 10.1261/rna.065482.117, PMID: 30012570 PMC6140458

[ref42] Shen T. Rui B. Zhou H. Zhang X. Yi Y. Wen H.. (2013). Metabolic flux ratio analysis and multi-objective optimization revealed a globally conserved and coordinated metabolic response of *E. coli* to paraquat-induced oxidative stress. Mol. BioSyst. 9, 121–132. 10.1039/C2MB25285F, PMID: 23128557

[ref43] Shimizu K. (2016). Metabolic regulation and coordination of the metabolism in bacteria in response to a variety of growth conditions. Adv. Biochem. Eng. Biotechnol. 155, 1–54. 10.1007/10_2015_320, PMID: 25712586

[ref44] Slauch J. M. (2011). How does the oxidative burst of macrophages kill bacteria? Still an open question. Mol. Microbiol. 80, 580–583. 10.1111/j.1365-2958.2011.07612.x, PMID: 21375590 PMC3109634

[ref45] Starzyk R. (1984). tRNA base modifications and gene regulation. Trends Biochem. Sci. 9, 333–334. 10.1016/0968-0004(84)90053-7

[ref46] Steiner R. E. Kyle A. M. Ibba M. (2019). Oxidation of phenylalanyl-tRNA synthetase positively regulates translational quality control. Proc. Natl. Acad. Sci. U. S. A. 116, 10058–10063. 10.1073/pnas.1901634116, PMID: 31036643 PMC6525502

[ref47] Subramaniam A. R. Deloughery A. Bradshaw N. Chen Y. O’Shea E. Losick R.. (2013a). A serine sensor for multicellularity in a bacterium. eLife 2:e01501. 10.7554/eLife.01501, PMID: 24347549 PMC3862929

[ref48] Subramaniam A. R. Pan T. Cluzel P. (2013b). Environmental perturbations lift the degeneracy of the genetic code to regulate protein levels in bacteria. Proc. Natl. Acad. Sci. U. S. A. 110, 2419–2424. 10.1073/pnas.1211077110, PMID: 23277573 PMC3568297

[ref49] Subramaniam A. R. Zid B. M. O’Shea E. K. (2014). An integrated approach reveals regulatory controls on bacterial translation elongation. Cell 159, 1200–1211. 10.1016/j.cell.2014.10.043, PMID: 25416955 PMC4243059

[ref50] Svenningsen S. L. Kongstad M. Stenum T. S. Muñoz-Gómez A. J. Sørensen M. A. (2017). Transfer RNA is highly unstable during early amino acid starvation in *Escherichia coli*. Nucleic Acids Res. 45, 793–804. 10.1093/nar/gkw1169, PMID: 27903898 PMC5314770

[ref51] Wiegand I. Hilpert K. Hancock R. E. W. (2008). Agar and broth dilution methods to determine the minimal inhibitory concentration (MIC) of antimicrobial substances. Nat. Protoc. 3, 163–175. 10.1038/nprot.2007.521, PMID: 18274517

[ref52] Willi J. Küpfer P. Evéquoz D. Fernandez G. Katz A. Leumann C.. (2018). Oxidative stress damages rRNA inside the ribosome and differentially affects the catalytic center. Nucleic Acids Res. 46, 1945–1957. 10.1093/nar/gkx1308, PMID: 29309687 PMC5829716

[ref53] Wu J. Fan Y. Ling J. (2014). Mechanism of oxidant-induced mistranslation by threonyl-tRNA synthetase. Nucleic Acids Res. 42, 6523–6531. 10.1093/nar/gku271, PMID: 24744241 PMC4041444

[ref54] Wu Y. Vulić M. Keren I. Lewis K. (2012). Role of oxidative stress in persister tolerance. Antimicrob. Agents Chemother. 56, 4922–4926. 10.1128/AAC.00921-12, PMID: 22777047 PMC3421885

[ref55] Yamamoto H. Wittek D. Gupta R. Qin B. Ueda T. Krause R.. (2016). 70S-scanning initiation is a novel and frequent initiation mode of ribosomal translation in bacteria. Proc. Natl. Acad. Sci. U. S. A. 113, E1180–E1189. 10.1073/pnas.1524554113, PMID: 26888283 PMC4780633

[ref56] Yutthanasirikul R. Nagano T. Jimbo H. Hihara Y. Kanamori T. Ueda T.. (2016). Oxidation of a cysteine residue in elongation factor EF-Tu reversibly inhibits translation in the cyanobacterium *Synechocystis* sp. PCC 6803. J. Biol. Chem. 291, 5860–5870. 10.1074/jbc.M115.706424, PMID: 26786107 PMC4786720

[ref57] Zhong J. Xiao C. Gu W. Du G. Sun X. He Q.. (2015). Transfer RNAs mediate the rapid adaptation of *Escherichia coli* to oxidative stress. PLoS Genet. 11:e1005302. 10.1371/journal.pgen.1005302, PMID: 26090660 PMC4474833

[ref58] Zhu M. Dai X. (2019). Maintenance of translational elongation rate underlies the survival of *Escherichia coli* during oxidative stress. Nucleic Acids Res. 47, 7592–7604. 10.1093/nar/gkz467, PMID: 31131413 PMC6698664

