# Supplementary material for: Modulation of Escherichia coli Translation by the Specific Inactivation of tRNAGly Under Oxidative Stress
Source: Front Genet. 2020 Aug 18;11:856. doi: 10.3389/fgene.2020.00856 (PMC7461829; doi:10.3389/fgene.2020.00856)
Supplement: Supplementary file 1 [file Data_Sheet_1.PDF]

Supplementary Material

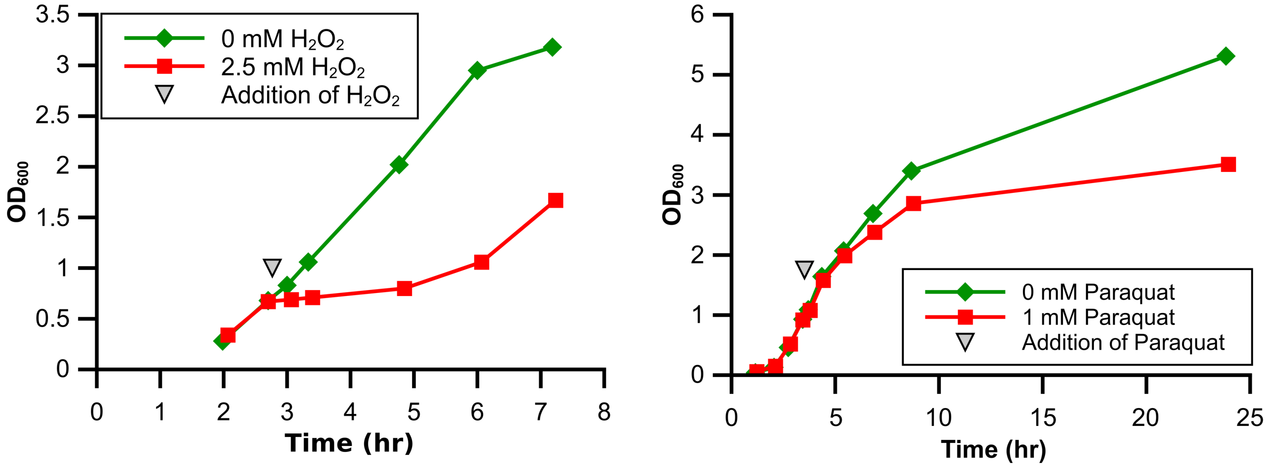

Supplementary figure 1. Effect of H_2O_2 and paraquat on growth curves of *E. coli* cultures. *E. coli* was cultured on LB at 37 ºC and shaking of 225 rpm. At OD_{600} of ~0.8-0.9 H_2O_2 or paraquat was added to cultures. At determined times samples were taken to meassure OD_{600} values. Figure shows single representative curves. Green diamonds (◆): control flask, red squares (■): stress flask, gray triangle (▼): addition of stressor.

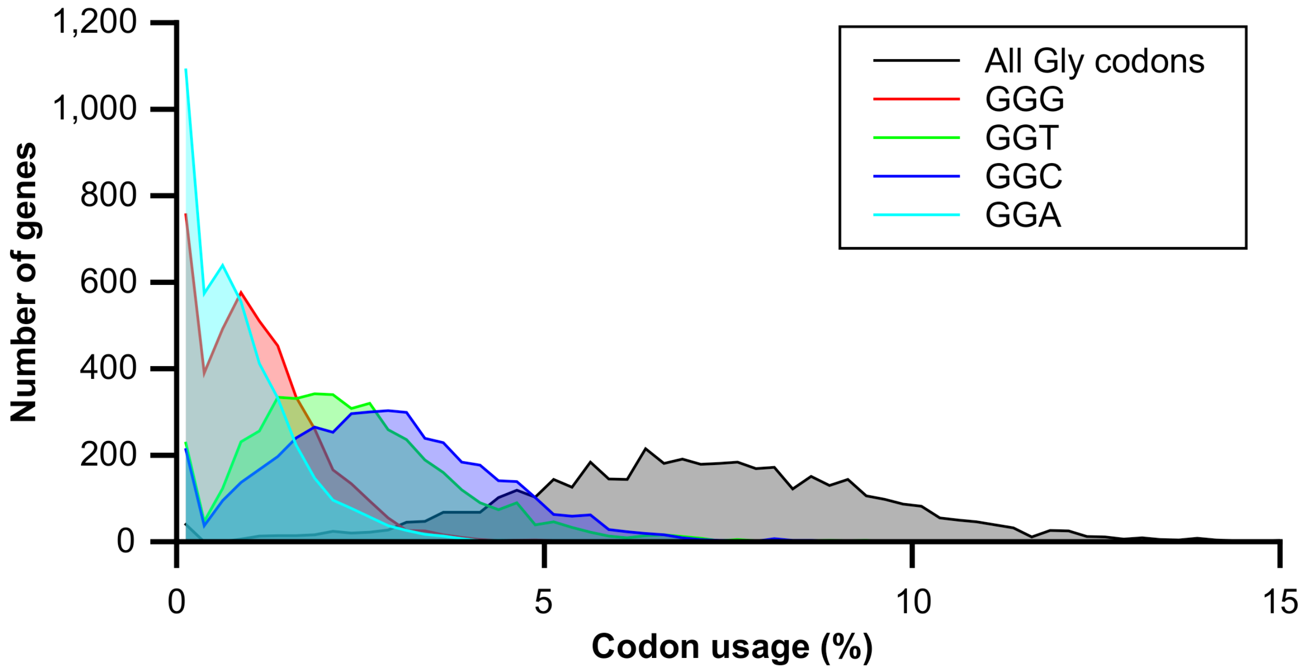

Supplementary figure 2. Usage of Gly codons in the genome of *E. coli* K12 MG1655. Figure shows a histogram representing the percentage of Gly codons used by genes annotated in the *E. coli* K-12 MG1655 genome sequence. Cyan: usage of GGA codons, purple: usage of GGC codons, green: usage of GGT codons, red: usage of GGG codons, black: usage of any Gly codon.

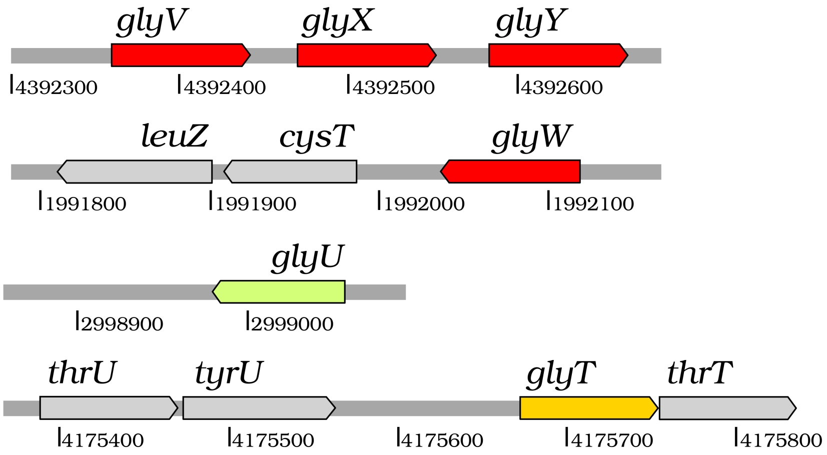

Supplementary figure 3. Genome context of tRNA^{Gly} genes. Schematic representation of the genome context of genes coding for tRNA. Genes for tRNAs with GCC anticodon are painted in red, CCC anticodon in green and UCC anticodon in orange.

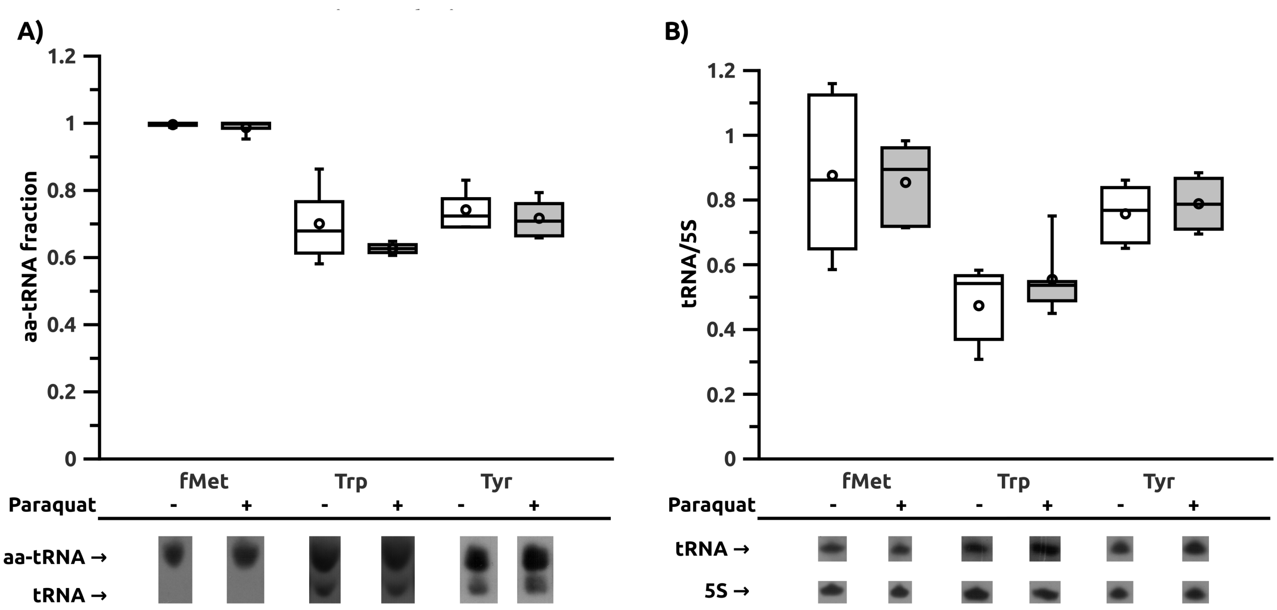

Supplementary figure 4. Effect of paraquat on the levels and aminoacylation of tRNA^{fMet}, tRNA^{Trp} and tRNA^{Tyr}. A) *In vivo* levels of aminoacylation of tRNA^{fMet}, tRNA^{Trp} and tRNA^{Tyr} in *E. coli* cell collected before (white bars) or 30 min after stress by 1 mM paraquat (gray bars). 3' terminal nucleotide of RNAs was eliminated by oxidation with sodium periodate followed by β-elimination and analyzed by Northern blot. Two not resolved bands were observed for tRNA^{Tyr}. These probably derives from the two tRNA^{Tyr} isotypes that have the same anticodon and a single nucleotide substitution (T/C) located at the variable arm. As bands were not resolved, these were considered as a single specie for all calculations. Thus, the reported level corresponds to the total tRNA^{Tyr} aminoacylation. (n=4). **B)** Effect of paraquat on the levels of tRNA as quantified by Northern blot of total RNA purified from samples collected before (white bars) or 30 min after 1 mM paraquat (gray bars) was added to *E. coli* cells. (n=5) Data in both graphs showed no significant differences using a two-tailed T-Student's test. In both box graphs top, middle and bottom lines of the box represent 25, 50 and 75 % of the population. Whiskers represent the maximum and minimum values and the mean is represented by a circle.

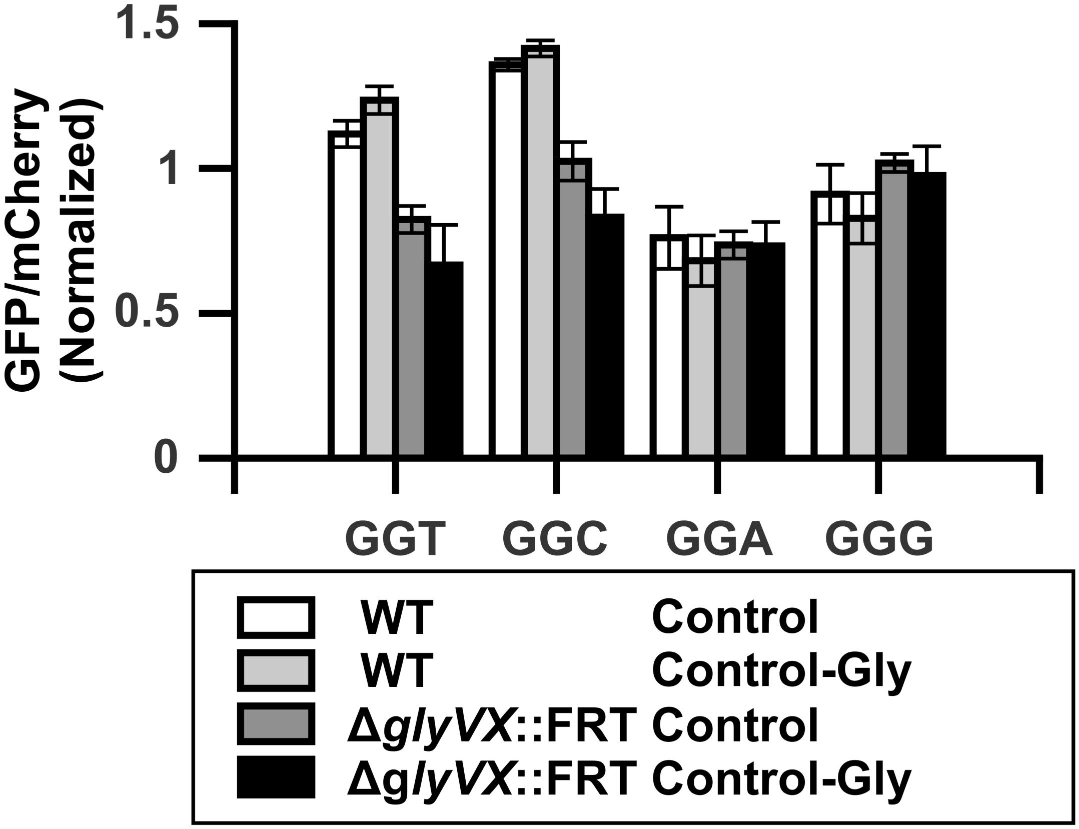

Supplementary figure 5. Effects of *glyVX* deletion on the translation of Gly codons. Figure shows GFP fluorescence normalized by fluorescence of mCherry in diverse strains and conditions. Data was additionally normalized dividing by the GFP/mCherry ratio of the control strain (reporter S1, without additional codons). The same reporters as in Figure 2 were transformed in WT and Δ*glyVX*::FRT *E. coli* K12 MG1655 strain. GFP/mCherry fluorescence ratios were measured under control (white for WT or dark gray for Δ*glyVX*::FRT strains) or control plus glycine (light gray for WT or black for Δ*glyVX*::FRT strains) conditions. Data was normalized by the fluorescence ratio found in the strain carrying the reporter with no additional codons. Data of WT strains is the same as in Figure 2, added here for comparison.

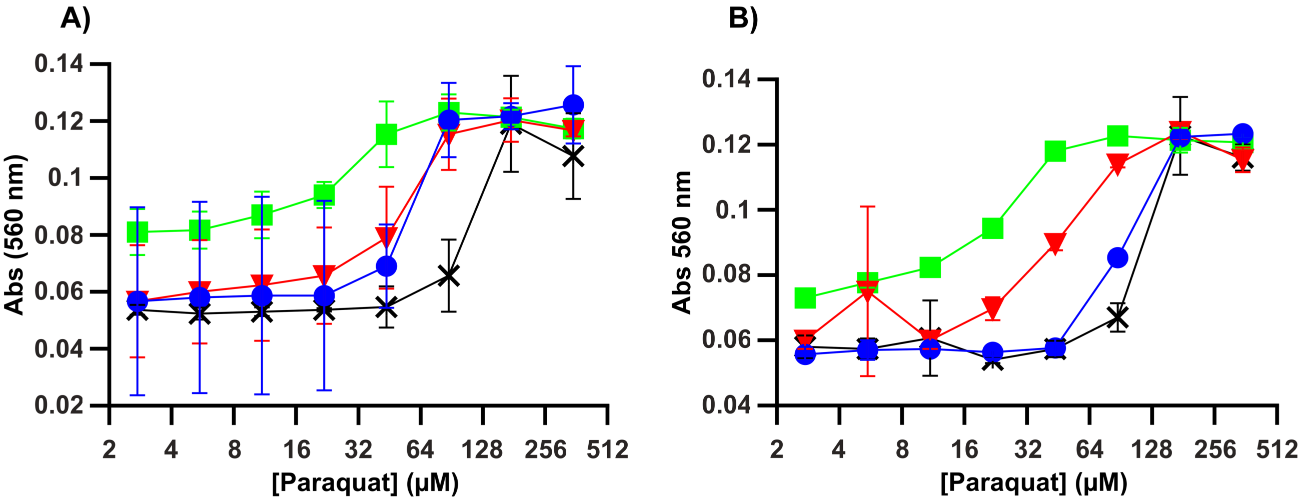

Supplementary figure 6. Inhibition of Consumption of carbohydrates under oxidative stress. Each strain was cultured in M9 media supplemented with branched amino acids, Gly, and the corresponding carbohydrate. Media also contained phenol red as an pH indicator and increasing concentration of paraquat. After 12 hrs incubation at 37 ºC cultures were pelleted and absorbance of the supernatant was measured at 560 nm. Higher absorbance indicates higher pH (lower fermentation of the carbohydrate). Figure shows experiments for fermentation of **A)** galactose and **B)** glucose. tRNA$^{Gly}_{CCC}$: green squares, tRNA$^{Gly}_{GCC}$: red triangles, tRNA$^{Gly}_{UCC}$: blue circles, empty pKK223-3 plasmid: black crosses.

Table S3. Oligonucleotides used for Northern blot experiments and purification of specific tRNAs

Target RNA	Anticodón	Probe name	Sequence (5' to 3')[a]	Concentration for northern (nM)	Hybridization and washing temperature (°C)
$tRNA^{Gly}{}_{U}$	CCC	B-SONglyU	B-GAATCGAACCCTCGTATAGAGCTTGGG	37	43
$tRNA^{Gly}{}_{T}$	UCC	B-SONglyT	B-GAATCGAACCCGCATCATCAGCTTGG	37	43
$tRNA^{Gly}{}_{V}$, $tRNA^{Gly}{}_{X}$, $tRNA^{Gly}{}_{Y}$	GCC	B-SONglyV	B-GACTCGAACTCGCGACCCCGACCTTGGC	2,5	43
$tRNA^{Trp}$	CCA	B-SONTrp	B-TGGAGACCGGTGCTCTACCAATT	30	38.5
$tRNA^{fMet}$	CAU	B-SONfMet	B-TATGAGCCCGACGAGCTACCAG	30	43
$tRNA^{Tyr}$	GUA	B-SONTyr	B-TACAGTCTGCTCCCTTTGGCCG	30	44
RNA 5S		B-SONRNA5S	B-TTCTGAGTTCGGCATGGGGTCAGGTGG	0,6	
$tRNA^{Gly}{}_{U}$	CCC	SON_glyU	GCGGGCGAAGGGAATCGAA	12	43
$tRNA^{Gly}{}_{T}$	CCC	SON_glyT	GCGGGCAGCGGGAATCGAA	12	43
$tRNA^{Trp}$	CCA	SON_Trp	TGGCAGGGGCGGAGAGACTCGAA	10	38.5
$tRNA^{fMet}$	CAU	SON_fMet	TGGTTGCGGGGGCCGGATTTGAA	10	43
$tRNA^{Tyr}$	GUA	SON_Tyr	TGGTGGTGGGGGAAGGATTCGAA	10	44

[a] "B" indicates a biotin added at the 5' extreme of the oligonucleotide

Table S4. Concentration of amino acids used for aminoacylation experiments

Amino acid	Concentration[a]
Ala	100 µM
Arg	100 µM
Gly	100 µM
Ile	100 µM
Leu	100 µM
Lys	50 µM
Phe	50 µM
Pro	100 µM
Tyr	25 µM
Val	25 µM

[a] Concentrations correspond to total amino acid concentration including both normal and radioactive amino acid

Table S5. Oligonucleotides used for cloning tetra-codon repeats in pBAD30SFIT.

Clone	Repeated codon	Sequence (5’ to 3’) Forward primer	Reverse primer
pBAD30SFIT/40>Gly|GGT|	Gly GGT	TCGAGGGTGGTGGTGGTA	CTAGTACCACCACCACCC
pBAD30SFIT/41>Gly|GGC|	Gly GGC	TCGAGGGCGGCGGCGGCA	CTAGTGCCGCCGCCGCCC
pBAD30SFIT/42>Gly|GGA|	Gly GGA	TCGAGGGAGGAGGAGGAA	CTAGTTCCTCCTCCTCCC
pBAD30SFIT/43>Gly|GGG|	Gly GGG	TCGAGGGGGGGGGGGGGA	CTAGTCCCCCCCCCCCCC

Table S6. Oligonucleotides used for cloning WT and mutant *narJ* as well as an internal hairpin in pBAD30SFIT.

Clone	Oligonucleotide (5’ to 3’) [a, b]	Note
NarJ_EcoRI_5'_Fw	CCGGAATTCAGGAGGAATTTACCATGATCGAACTCGTG ATT	WT oligonucleotide
NarJ_XhoI_3'_GGA_Rv	CCGCTCGAGGTGCTG**TCCTCC**GGTGGTGATATTCA	WT oligonucleotide
NarJ_XhoI_3'_GGC_Rv	CCGCTCGAGGTGCTG**GCCGCC**GGTGGTGATATTCA	Mutant oligonucleótide
Str_Yam_Fw	GATCTGATCCAAACAAAACAAAAC**GGGGACCCC**TTGC **GGGGTCCCC**AAAACA	hairpin oligonucleotide
Str_Yam_Rv	CATGTGTTTT**GGGGACCCC**GCAA**GGGGTCCCC**GTTTT GTTTTGTTTGGATCA	hairpin oligonucleotide

[a] Underlining indicates restriction sites.

[b] Mutated site in *narJ* and double helix in inserted hairpin are highlighted in bold letters

Table S7. Oligonucleotides used in cloning of tRNA genes in pKK223-3 plasmid

Oligonucleotide	Sequence[a]
tRNAglyU5	AATTCATCTCGAAGCGGGCGTAGTTCAATGGTAGAACGAGAGCTTCCC
tRNAglyU5c	GAGCTTGGGAAGCTCTCGTTCTACCATTGAACTACGCCCGCTTCGAGATG
tRNAglyU3	AAGCTCTATACGAGGGTTCGATTCCCTTCGCCCGCTCCAATTTATCTA
tRNAglyU3c	AGCTTTGATAAATTGGAGCGGGCGAAGGGAATCGAACCCTCGTATA
tRNAglyT5	AATTCTCCAGGATGCGGGCATCGTATAATGGCTATTACCTCAGCCTTC
tRNAglyT5c	AGCTTGGAAGGCTGAGGTAATAGCCATTATACGATGCCCGCATCCTGGAG
tRNAglyT3	CAAGCTGATGATGCGGGTTCGATTCCCGCTGCCCGCTCCAAGATGTGCA
tRNAglyT3c	AGCTTGCACATCTTGGAGCGGGCAGCGGGAATCGAACCCGCATCATC
tRNAglyV5	AATTCAGTAGTACGCGGGAATAGCTCAGTTGGTAGAGCACGACCTTGC
tRNAglyV5c	ACCTTGGCAAGGTCGTGCTCTACCAACTGAGCTATTCCCGCGTACTACTG
tRNAglyV3	CAAGGTCGGGGTCGCGAGTTCGAGTCTCGTTTCCCGCTCCAAAATTTGA
tRNAglyVc	AGCTTCAAATTTTGGAGCGGGAAACGAGACTCGAACTCGCGACCCCG
tRNAtyrV5	AATTCTTACCCCTGGTGGGGTTCCCGAGCGGCCAAAGGGAGCAGACTGTAAAT
tRNAtyrV5c	CGGCAGATTTACAGTCTGCTCCCTTTGGCCGCTCGGGAACCCCACCAGGGGTAAG
tRNAtyrV3	CTGCCGTCATCGACTTCGAAGGTTCGAATCCTTCCCCCACCACCATCACTTTCA
tRNAtyrVc	AGCTTGAAAGTGATGGTGGTGGGGGAAGGATTCGAACCTTCGAAGTCGATGA

[a] Underlining indicates EcoRI and HindIII sites.

Table S8. Oligonucleotides used for deleting *glyVX* genes in *E. coli* K12 MG1655

Oligonucleotide	Sequence[a]
glyV (H1+P1)	GCAGAAATGCGAAAATTACGAAAGCAAAATTAAGTAGTACgtgcaggctggagctgcttc
glyX (H2+P2)	CCCGCTTGGGTGGTCTGTGCCTTACAGCACTTTCAAATTTcatatgaatatcctccttag

[a] Lowercase indicate the region that anneals to the 5' or 3' end of the antibiotic resistance cassette used for the mutagenesis.
